# Environmental Exposure to Endocrine-Disrupting Chemicals and SARS-CoV-2 Infection History Among Blood Donors

**DOI:** 10.3390/life16081379

**Published:** 2026-08-21

**Authors:** Wioletta Ratajczak-Wrona, Katarzyna Bielawska, Jolanta Wrobel, Agnieszka Zebrowska, Kierek Zofia, Magdalena Pabian, Natalia Radziwon, Krzysztof Matuk, Jakub Rudzinski, Wojciech Miltyk, Piotr Radziwon, Barbara Pucelik, Bartlomiej Zalewski, Zbigniew Wochynski, Ewa Jablonska

**Affiliations:** 1 Department of Immunology, Medical University of Bialystok, 15-269 Bialystok, Poland; 2 Department of Pharmaceutical and Biopharmaceutical Analysis, Faculty of Pharmacy with the Division of Laboratory Medicine, Medical University of Bialystok, 15-089 Bialystok, Poland; 3 Regional Centre for Transfusion Medicine, 15-269 Bialystok, Poland; 4 Imago Sp. z o.o., 15-688 Bialystok, Poland; 5 Laboratory of Environmental Chemoinformatics, Faculty of Chemistry, University of Gdansk, 80-308 Gdansk, Poland; 6 Lukasiewicz Research Network, Krakow Institute of Technology, 30-418 Krakow, Poland; 7 SORNO Medical and Rehabilitation Center, 05-082 Stare Babice, Poland; 8 Department of Air Transport Safety, Polish Air Force University, 08-521 Deblin, Poland

**Keywords:** EDCs, COVID-19, biomonitoring, environmental exposure, SARS-CoV-2, blood donors, chemical mixtures

## Abstract

**Objectives:** The present study evaluated the association between plasma concentrations of selected endocrine-disrupting chemicals (EDCs)—bisphenol A (BPA), methylparaben (MeP), propylparaben (PrP), 4-nonylphenol (4-NP), 4-octylphenol (4-OP), bis(2-ethylhexyl) phthalate (DEHP), and imazalil (IMZ)—and prior coronavirus disease 2019 (COVID-19) status. **Methods:** Plasma EDC levels were quantified via gas chromatography–mass spectrometry in 262 voluntary blood donors (121 convalescents and 141 controls) at the Regional Centre for Transfusion Medicine in Bialystok, Poland. **Results:** The cohort exhibited widespread environmental exposure to all selected EDCs (BPA: 1.14 ng/mL, MeP: 1.20 ng/mL, PrP: 1.38 ng/mL, 4-NP: 1.90 ng/mL, 4-OP: 0.57 ng/mL, DEHP: 49.68 ng/mL, IMZ: 20.06 ng/mL). Convalescents had significantly higher concentrations of MeP, PrP, 4-OP, and 4-NP. Logistic regression revealed that MeP, PrP, and 4-NP were significantly associated with prior COVID-19 status, increasing infection odds by 80%, 70%, and 63%, respectively. While 4-OP was strongly associated with prior COVID-19 status (odds ratio > 300 per unit change and 1.79 per 0.1 unit change), the wide confidence interval for the unit change estimate requires cautious interpretation. **Conclusions:** These findings suggest a strong link between selected paraben and alkylphenol exposure and prior COVID-19 status in blood donors. Additionally, the high IMZ levels highlight the need for urgent evaluation of its safety and environmental prevalence.

## 1. Introduction

The historical framework for evaluating endocrine-disrupting chemicals (EDCs) originated 35 years ago at the Wingspread Conference in Racine, WI, USA (26–28 July 1991), where a group of 21 experts first assessed the impact of industrial and agricultural chemicals on the endocrine systems in humans and wildlife. These compounds, capable of disrupting endocrine activity by mimicking, blocking, or interfering with hormonal activity, were classified as EDCs [1]. Mechanistically, EDCs act as ligands interacting with nuclear receptors—including estrogen receptors, androgen receptors, thyroid hormone receptors, peroxisome proliferator-activated receptors, and aryl hydrocarbon receptors. Through this binding, EDCs block endogenous ligands, alter receptor-dependent intracellular processes, modulate the activity of ion channels, and drive epigenetic changes in exposed cells [2,3,4].

EDCs are heterogeneous substances that serve various commercial and industrial applications. These include bisphenol A (BPA), an organic phenol widely used to manufacture plastics, particularly polycarbonates and epoxy resins for food and beverage containers, reusable bottles, and thermal paper; parabens, such as methylparaben (MeP) and propylparaben (PrP), which are p-hydroxybenzoic acid esters used as preservatives in cosmetics, personal care products, pharmaceuticals, and processed foods due to their antimicrobial properties; alkylphenols, such as 4-nonylphenol (4-NP) and 4-octylphenol (4-OP), which are found in paints, herbicides, pesticides, nonionic detergents, cosmetics, plastic containers, and composite materials; bis(2-ethylhexyl) phthalate (DEHP), a phthalate used as a plasticizer in plastics, particularly polyvinyl chloride, and as a solvent in resins, adhesives, and varnishes; and imazalil (IMZ), a fungicide from the imidazole derivative group widely used in agriculture to protect fruits, vegetables, and wheat from mold and in veterinary medicine as a topical antifungal agent [5,6,7,8,9,10,11,12,13]. The ubiquitous environmental presence of EDCs promotes their absorption by the body, raising safety concerns for products containing them. Research since the 1990s into the harmful effects of DEHP resulted in a ban on its use in children’s products like bottles and pacifiers; in 2017, it was added to the REACH list of substances of a very great concern. The use of BPA in children’s products is also banned, and several European countries require manufacturers to use substitutes. Regulatory standards set by the European Union and the Food and Drug Administration (FDA) also restrict MeP, PrP, 4-NP, and 4-OP. Despite the restrictions, EDCs accumulate in the body and environment, where they can convert into more toxic derivatives [14,15,16].

Scientific reports link EDCs to the pathogenesis of hormone-dependent diseases, including hypothyroidism, obesity, type 2 diabetes, and prostate and breast cancers. Studies also show clear links between EDC exposure and cardiovascular, respiratory, urinary, nervous, and immunological diseases. It is hypothesized that by altering immune cell development and effector functions, these compounds disrupt antibacterial and antiviral defense mechanisms [6,7,17,18,19,20,21,22].

A key element in EDC-mediated immune regulation is the receptor for activated C kinase 1 (RACK1). RACK1 regulates numerous signaling pathways vital to cellular functions. Previous studies suggest that modulation of RACK1 by EDCs disrupts signaling pathways responsible for immune cell activation and cytokine production. Consequently, this can impair the antiviral response of the host [23,24,25,26,27,28,29,30].

Evidence confirms the critical role of EDCs in promoting the development of influenza A virus subtype H1N1 (*A/H1N1*) infection in an animal model. Similar to acute respiratory distress syndrome caused by SARS-CoV-2 (coronavirus disease 2019—COVID-19), H1N1 comorbidities include diabetes, kidney disease, and cardiovascular diseases [31,32,33]. Long-term exposure to EDCs has been shown to significantly drive the onset and progression of these comorbidities [34].

On the other hand, the literature suggests that environmental pollution may have exacerbated the severity of the COVID-19 pandemic by disrupting immune system regulation. In addition, air pollution impairs male reproductive health, contributing to the global decline in semen quality observed over recent decades. Importantly, semen quality has been proposed as a potential indicator of SARS-CoV-2 susceptibility in polluted areas, suggesting environmental pollutants increase vulnerability to COVID-19 and partially explain the higher incidence and severity of the disease in men compared with women [35,36].

Furthermore, a recent computational systems biology study investigated the relationship between EDCs and COVID-19 severity. The analysis identified the Th17 and AGE/RAGE signaling pathways as the principal molecular mechanisms through which EDCs may exacerbate COVID-19 [37].

COVID-19, a pandemic disease caused by SARS-CoV-2 infection, transmits mainly via droplets. This was first detected in December 2019 in Wuhan, China. SARS-CoV-2 is an enveloped virus containing single-stranded, positive-sense RNA that causes respiratory symptoms. It is composed of four structural proteins: spike (S) protein (mediating the interaction of virus particles with the receptor), envelope (E) protein (driving virion formation), membrane (M) protein (shaping the viral envelope), and nucleocapsid (N) protein (protecting the RNA molecule and modifying cellular processes and virus replication). SARS-CoV-2 enters host cells via endocytosis through the binding of the S protein to the human angiotensin-converting enzyme 2 receptor [38,39].

Given the similarities between COVID-19 and influenza, our primary objective was to assess whether higher exposure to EDCs is associated with prior COVID-19 status. Biological material was collected from voluntary blood donors between 1 May and 30 July 2021. We pursued the study goal by: (1) measuring the concentrations of selected EDCs (BPA, MeP, PrP, 4-NP, 4-OP, DEHP, and IMZ) in blood plasma collected from donors—both those previously infected with SARS-CoV-2 (convalescents) and those without a history of COVID-19; and (2) applying logistic regression models to estimate how a one-unit change in the concentration of individual EDCs affects infection risk, using prior COVID-19 status as the representative clinical outcome.

## 2. Methods

### 2.1. Study Population

This study involved 121 convalescent voluntary blood donors (11 women and 110 men) with a history of SARS-CoV-2 infection confirmed by a polymerase chain reaction (PCR) or antigen testing, from the Regional Centre for Transfusion Medicine in Bialystok, Poland. The donors were recruited following a detailed medical interview and a standard physical examination. Prior SARS-CoV-2 infection status was verified using medical records. None of the donors required hospitalization, as all experienced mild COVID-19. At the time of blood donation, no donors presented with known major comorbidities. All donors were White/Caucasian individuals from the Podlaskie Voivodeship in northeastern Poland, aged between 20 and 65 years.

The control group consisted of 141 voluntary blood donors (37 women and 104 men) from the same center, with no history of SARS-CoV-2 infection, confirmed by PCR or antigen testing. The donors were recruited after a detailed interview and a standard physical examination, with ages ranging from 20 to 65 years. Both the study group and control group donors were informed about the purpose of the study and provided written informed consent.

This study was approved by the Ethics Committee of the Medical University of Bialystok (No. APK.002.102.2022).

Following consent of donors, a 2 mL blood sample was collected from the ulnar vein into an anticoagulant. To isolate plasma, samples were centrifuged at 2000× *g* for 5 min. Plasma aliquots were carefully transferred to Eppendorf tubes and stored at −80 °C until analysis in 2022. All biological materials were collected between 1 May and 30 July 2021.

To minimize potential contamination with bisphenol leaching from the plastic Eppendorf tubes, all blood samples were processed immediately after collection, and the corresponding analyses were performed as soon as possible. Identical sample collection, handling, storage, and analytical procedures were applied to all donors to ensure that any potential background contamination affected all samples equally.

### 2.2. Chemicals, Standards, and Analytical Quality Control

The analytical standards of EDCs (BPA, MeP, PrP, 4-NP, 4-OP, DEHP, IMZ, carbon-labeled internal standard BPA (BPA^13^C_12_) and the silylation derivatization reagent BSTFA:TMCS (99:1) were obtained from Sigma-Aldrich (St Louis, MO, USA). All solvents used in this study were of gas chromatography–mass spectrometry (GC–MS) grade and were purchased from Merck (Darmstadt, Germany).

Stock solutions of individual EDCs (1 mg/mL) were prepared in methanol and stored at −20 °C in screw-capped glass vials. Working solutions were prepared daily from these stocks by appropriate dilution. All glassware was pre-rinsed with methanol, and all plastics consisted of high-quality polypropylene to prevent exogenous bisphenol contamination.

The daily batches of study and quality control samples were selected randomly. In addition, the analysis of each daily batch was accompanied by the analysis of three procedural blank samples. The mean blank value was calculated from these blanks, and the concentrations of individual EDCs were corrected by subtracting the corresponding mean blank value. The order of chromatographic analysis of the prepared samples was also randomized. The validation of the analytical methods for EDC determination did not include the assessment of analyte recovery or intra-assay and inter-assay precision.

### 2.3. Determination of EDCs in Plasma Samples

For the GC–MS determination of EDCs (BPA, MeP, PrP, 4-NP, 4-OP, and DEHP), 50 µL of plasma samples was spiked with internal standard (BPA ^13^C_12_), followed by liquid–liquid extraction with 500 µL of ethyl acetate. After vortexing for 15 min, samples were centrifuged for 10 min at 5000× *g* (4 °C). The supernatants were evaporated to dryness under argon gas. Finally, the dried residue was derivatized with 50 µL of BSTFA containing 1% TMCS at 70 °C for 30 min, and 1 µL of the resulting solution was injected into the GC–MS system operating in selected ion monitoring mode.

To isolate IMZ from plasma samples (50 µL), a dual liquid–liquid extraction with ethyl acetate (500 µL) was performed. After vortexing for 10 min, samples were centrifuged for 10 min at 5000× *g* (4 °C). The combined supernatants were evaporated to dryness under argon, and the dried residue was dissolved in 50 µL of ethyl acetate prior to a 1-μL sample injection.

Chromatographic separations were performed using a 7890A GC System coupled to a Q mass spectrometer (5975C VL MSD) (Agilent Technologies, Palo Alto, CA, USA) with an electron ionization source (ionization energy 70 eV). An HP-5ms capillary column (0.25 mm i.d., 0.25 µm film thickness, 30 m length; Agilent Technologies) with electronic pressure control and a split/splitless injector was used. Helium served as the carrier gas at a constant column flow rate of 1 mL/min. For derivatized samples, the injector operated at 280 °C in splitless mode. The initial column temperature was held at 70 °C (3 min), ramped at 15 °C/min to 150 °C, increased to 250 °C at 7 °C/min, and finally raised at 5 °C/min to 300 °C, where it was held for 15 min. The MSD detector acquisition parameters were as follows: transfer line temperature 275 °C and the MS source temperature 230 °C. Derivatized EDCs were detected by selected ion-monitoring GC/MS. The ions used were: m/z 209 for MeP, and m/z 193.0 and 252.0 for PrP; m/z 179.0 and 278.0 for 4-OP; m/z 179.0 and 292.0 for 4-NP; m/z 357.0 and 372.0 for BPA; m/z 149.0 and 167.0 for DEHP and m/z 369.0 and 384.0 for internal standard (BPA13C12) derivative.

Quantification was performed using an internal standard calibration curve. Linearity was achieved for all analytes, with correlation coefficients exceeding 0.996. The limit of quantitation (LOQ) was 0.1 ng/mL for MeP, 0.2 ng/mL for PrP, BPA, and DEHP, 0.5 ng/mL for 4-OP, and 1.0 ng/mL for 4-NP.

GC–MS analysis of IMZ was carried out using the following conditions: the column temperature was initially set at 120 °C for 1 min, increased by 10 °C/min to 190 °C (1 min), ramped at 5 °C/min to 250 °C (5 min), and finally raised at 10 °C/min to 300 °C, where it was held for 5 min. The split/splitless injector was maintained at 280 °C, the transfer line at 280 °C, and the source temperature at 230 °C. Helium was used as the carrier gas at a flow rate of 1 mL/min under splitless injection mode. Imazalil was detected by selected ion-monitoring GC/MS (m/z 173.0 and 215.0).

Quantitation was achieved using an external standard method, yielding a correlation coefficient (*r*^2^) above 0.998. The limit of detection for this method was 1 ng/mL, and the LOQ was 5 ng/mL.

### 2.4. Statistical Analysis

Statistical analyses were performed using R: A Language and Environment for Statistical Computing (version 4.4.2). Numerical variables are presented as mean ± standard deviation for normally distributed variables (age) or as median with interquartile range (IDR) for non-normally distributed variables (EDCs). Normality was evaluated using the Shapiro–Wilk test, with additional assessments of skewness and kurtosis. Homogeneity of variances was verified using Levene’s test. Differences between convalescents and controls were evaluated using Student’s *t*-test, Mann–Whitney *U* test, and Pearson’s chi-square test. To identify risk factors for prior COVID-19 status, logistic regression modeling was employed using a two-step approach: first, univariate models were built, followed by a multivariable assessment. To limit model overfitting relative to the number of outcome events, variables demonstrating a preliminary cutoff *p*-value of <0.25 in the univariate analysis were considered for inclusion in the multivariate model. A stepwise selection process was subsequently applied to finalize the selection. Fit of the multivariate model was assessed using Nagelkerke’s *R*^2^ and the Hosmer–Lemeshow test. Multicollinearity was evaluated through variance inflation factor (VIF) values, with all indicators remaining below 1.5, confirming low collinearity. As a sensitivity analysis, an additional multivariate model including all measured EDCs without variable selection was fitted.

Additionally, sensitivity univariate logistic regression analyses of 4-OP were performed: with alternative unit definition and excluding outliers with upper Tukey fence (Q3 + 1.5 × IQR) used as outlier cutoff.

To control for multiple comparisons, the Benjamini–Hochberg false discovery rate correction was applied to the primary group comparisons and univariable logistic regression analyses. The corrected results are presented in Supplementary Table S3.

Exploratory receiver operating characteristic (ROC) analysis was conducted to evaluate the ability of individual EDCs to predict prior COVID-19 status. The optimal cutoff value was determined using Youden’s index; this value should be considered hypothesis-generating rather than a clinically validated diagnostic threshold. Statistical significance was defined as *p* < 0.05 across all analyses.

## 3. Results

### 3.1. Characteristics of the Total Study Group and Comparison of Analyzed Parameters Between Convalescents and the Control Group

This study included 262 voluntary blood donors, of whom 81.7% were male. The mean age was 36.05 ± 9.67 years. The levels of EDCs in the total study group are presented in Table 1.

The convalescent cohort comprised 121 individuals (46.2%). Age and gender significantly differentiated the convalescent and control groups. Convalescents were on average 4 years older than the control group (mean difference [MD] = 4.32, 95% confidence interval [CI] [2.02; 6.63], *p* < 0.001). The proportion of male participants was 23% relatively higher among convalescents (90.0% vs. 73.8%) compared to the control group, which corresponds to a relative risk of 1.23 (95% CI [1.10; 1.38], *p* = 0.001).

**Figure 1:**
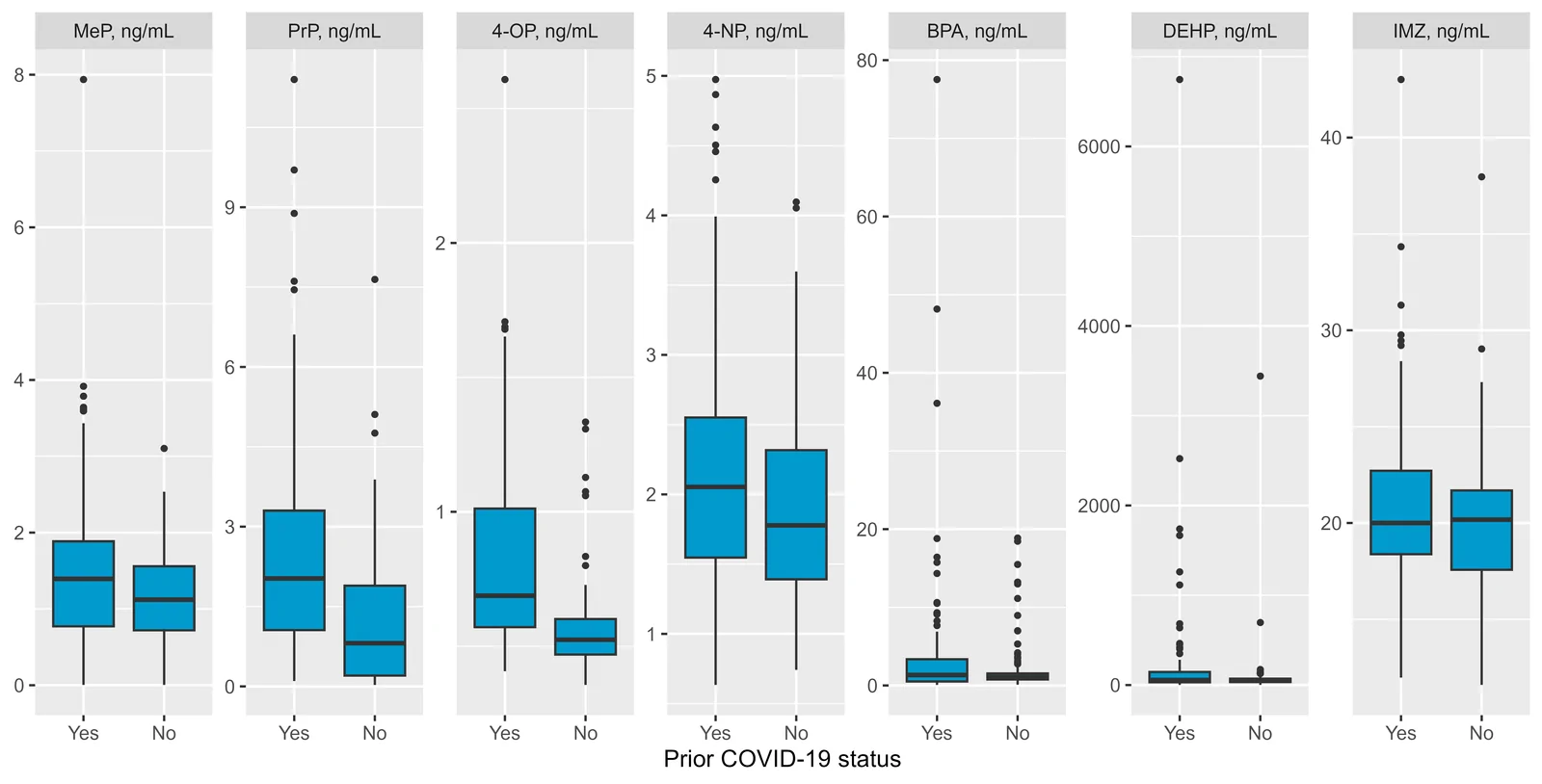
Boxplot charts presenting the concentrations of EDCs (BPA, MeP, PrP, 4-NP, 4-OP, DEHP, and IMZ; ng/mL) in convalescents (prior COVID-19 status: “Yes”) and the control group (prior COVID-19 status: “No”).

The plasma concentrations of MeP, PrP, 4-OP, and 4-NP differentiated the two groups, with significantly higher concentrations observed among convalescents (MeP: MD = 0.27 95% CI [0.08; 0.45]; *p* = 0.004, PrP: MD = 1.22, 95% CI [0.66; 1.32], *p* < 0.001; 4-OP: MD = 0.16, 95% CI [0.13; 0.23], *p* < 0.001; and 4-NP: MD = 0.27, 95% CI [0.07; 0.42], *p* = 0.007). Statistical differences in the concentrations of BPA, DEHP, and IMZ between convalescents and the control group were not observed (Table 1). Application of *p*-value corrections for multiple comparisons did not materially alter the results; all statistically significant associations remained significant after the adjustment (Supplementary Table S3). The distribution of EDCs in the two groups is presented in Figure 1.

### 3.2. EDC Concentrations Associated with Prior COVID-19 Status

Logistic regression was used to verify whether EDC concentrations were associated with prior COVID-19 status (Table 2), with gender and age included as covariates. Based on the univariate models, both gender and age correlated with prior SARS-CoV-2 infection. Male gender increased the odds of prior COVID-19 status nearly fourfold (odds ratio [OR] = 3.56, 95% CI [1.78; 7.66], *p* = 0.001), while each 1-year increase in age increased the odds by 5% (OR = 1.05, 95% CI [1.02; 1.08], *p* < 0.001). A one-unit increase in MeP concentration was associated with 80% higher odds (OR = 1.80, 95% CI [1.30; 2.55], *p* = 0.001). Similarly, a one-unit increase in PrP concentration correlated with 70% higher odds (OR = 1.70, 95% CI [1.40; 2.11], *p* < 0.001). A one-unit increase in 4-OP concentration was associated with over 300-fold higher odds (OR = 333.03, 95% CI [62.46; 2344.47], *p* < 0.001). Although 4-OP concentration showed the strongest association with prior COVID-19 status, the estimated effect size was accompanied by a very wide confidence interval, indicating marked statistical uncertainty that demands cautious interpretation. Furthermore, a one-unit increase in 4-NP was linked with 63% higher odds of prior COVID-19 status (OR = 1.63, 95% CI [1.19; 2.28], *p* = 0.003), while a one-unit increase in BPA was associated with a 7% higher odds (OR = 1.07, 95% CI [1.01; 1.16], *p* = 0.041). Other EDCs did not significantly impact the likelihood of a prior infection. Application of *p*-value corrections for the primary univariate logistic regression models did not alter the conclusions, and all statistically significant associations remained valid, except for the relationship between BPA and prior COVID-19 status (*p* = 0.053, Supplementary Table S1).

To assess the robustness of the association between 4-OP and prior COVID-19 status, sensitivity analyses were performed using alternative exposure scaling and after excluding extreme values; the association remained statistically significant across all analyses. When modeled per 0.1-unit increase in 4-OP, the OR was 1.79 (95% CI [1.51; 2.17], *p* < 0.001). Following the exclusion of potential outliers, the association for a 0.1-unit increase remained significant (OR = 2.57, 95% CI [1.95; 3.52], *p* < 0.001; Supplementary Table S2).

Multivariate model outcomes present adjusted ORs that account for gender, age, and relationships between the EDCs. In this model, gender and age remained significantly associated with prior COVID-19 status (*p* = 0.018 and *p* = 0.002, respectively). Additionally, a 0.1-unit increase in 4-OP concentration was associated with 68% higher odds of prior infection (OR = 1.68, 95% CI [1.33; 2.18], *p* < 0.001). A one-unit increase in IMZ was also found to be associated with over 8% higher odds of prior COVID-19 status (OR = 1.08, 95% CI [1.01; 1.17], *p* = 0.033). Multivariate model fit was verified with Nagelkerke R^2^ with the outcome of 41% and Hosmer and Lemeshow Goodness of Fit (GOF) Test with the outcome of *p* = 0.525, both indicating good model fit. VIF values were below 1.5, indicating low multicollinearity. In the sensitivity multivariable model including all measured EDCs without variable selection, 4-OP remained significantly associated with prior COVID-19 status, whereas IMZ and the remaining EDCs did not reach statistical significance (Supplementary Table S3).

### 3.3. Association Between EDC Levels and Prior COVID-19 Status

**Figure 2:**
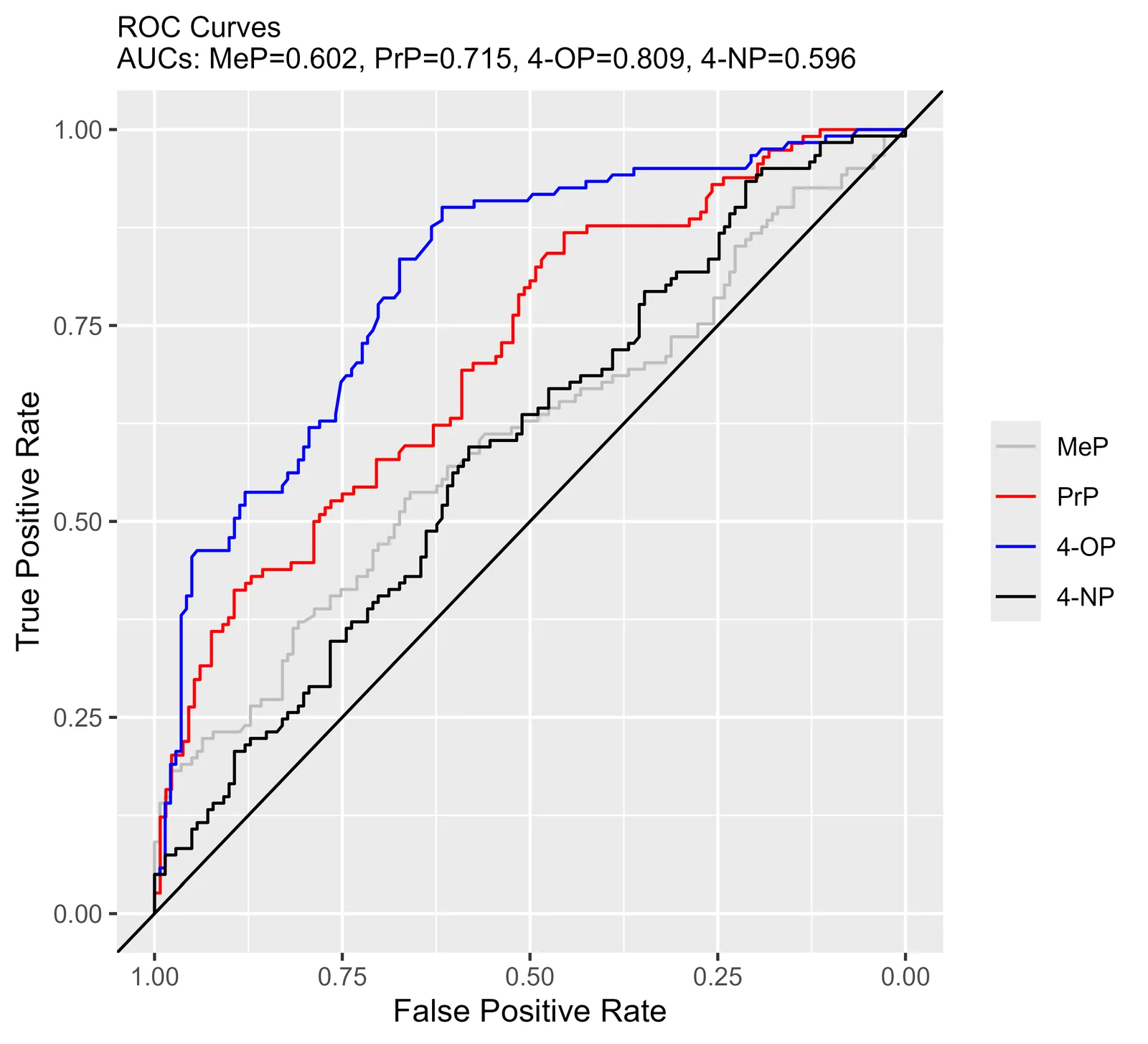
ROC curves for MeP, PrP, 4-OP and 4-NP in assessing the association between EDCs exposure and prior COVID-19 status.

Exploratory ROC analysis was performed to assess the discriminatory ability of EDCs for prior COVID-19 status. In the total study population, 4-OP demonstrated the highest effectiveness, with an area-under-the-curve (AUC) value of 0.809 (95% CI [0.755; 0.860]). The second-highest performance was confirmed for PrP with an AUC value of 0.715 (95% CI [0.645; 0.778]; Table 3). The corresponding ROC curves are presented in Figure 2.

**Figure 3:**
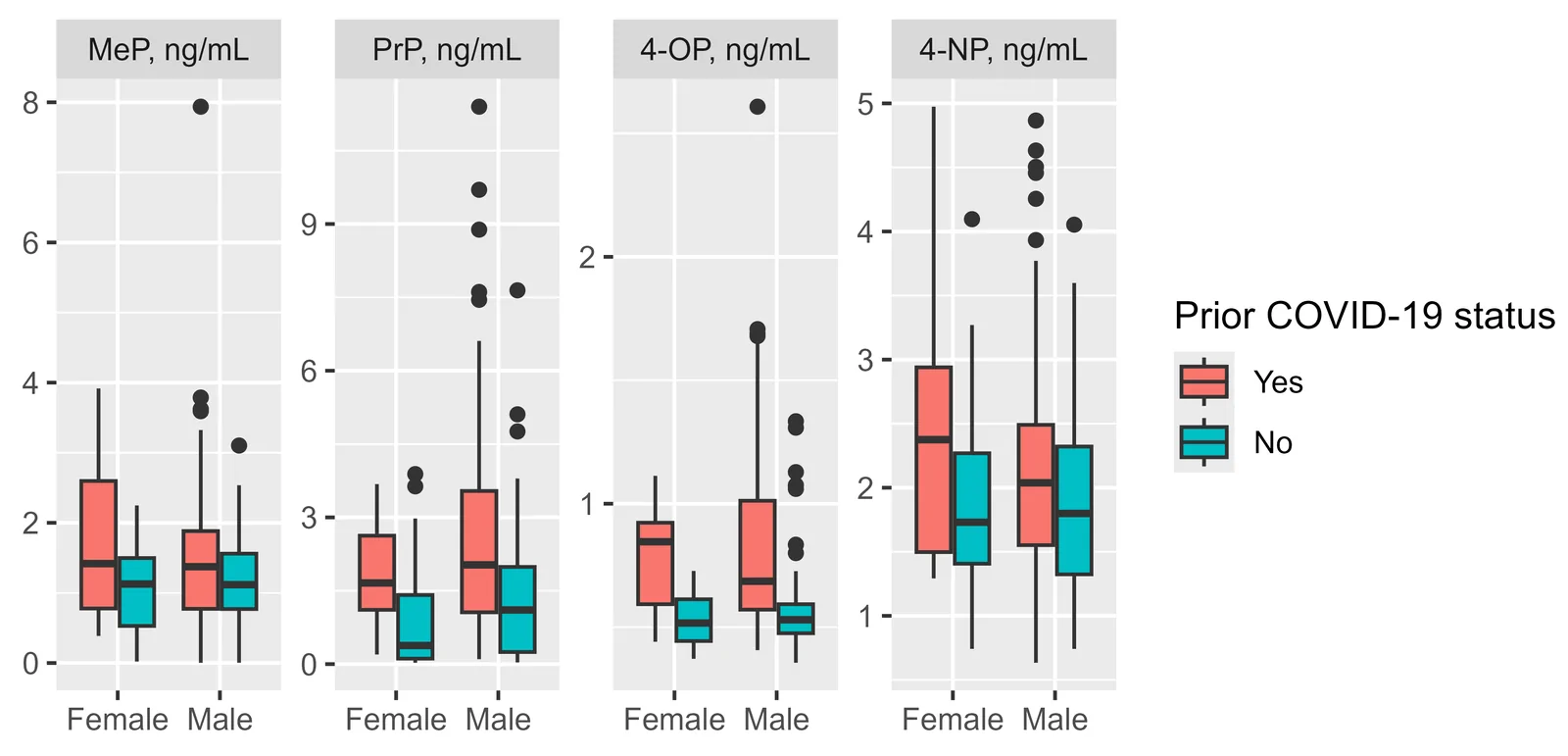
Boxplot charts presenting concentration of MeP, PrP, 4-OP and 4-NP in convalescents and control group, in additional split to gender (prior COVID-19 status: “Yes” and “No”).

ROC analysis was subsequently stratified by gender subgroups. The discriminatory ability for prior COVID-19 status was high for 4-OP and moderate for PrP exposure in both gender subgroups, consistent with the AUC values listed in Table 3. For PrP concentrations, the optimal cutoff values for predicting prior COVID-19 status differed between men and women. Among males, prior COVID-19 status was predicted by a PrP concentration above 2.39 ng/mL, yielding a sensitivity of 42% and a specificity of 91%, while the cutoff for females was 0.84 ng/mL with a sensitivity and specificity of 82% and 62%, respectively. Conversely, gender did not differentiate the optimal cutoff for 4-OP, which was 0.55 ng/mL among males (sensitivity: 91%, specificity: 60%) and 0.54 ng/mL among females (sensitivity: 91%, specificity: 65%). The distribution of PrP and 4-OP in split to convalescents and control group and by gender is presented as Figure 3.

## 4. Discussion

Our findings align with previous reports regarding the impact of age and sex on COVID-19 risk. Men demonstrated a nearly fourfold higher likelihood of prior infection, with the risk increasing by 5% with each additional year of age.

This study evaluated the potential impact of EDC exposure on prior SARS-CoV-2 infection status. Our results confirmed environmental exposure among donors from the Regional Centre for Transfusion Medicine in Bialystok, Poland, to all seven studied EDCs (BPA, MeP, PrP, 4-NP, 4-OP, DEHP, and IMZ). For six of these compounds, the mean plasma concentrations were similar to those reported previously [40,41,42,43,44]. The exception was IMZ, which exhibited a 300-fold higher plasma concentration than that observed in healthy Chinese volunteers [45]. The surprisingly high concentration of this fungicide in residents of northeastern Poland—a region characterized by intensive conventional agriculture—compared with individuals from both industrialized and agricultural areas of Jiangsu Province, China, indicates that pesticide application regulations, specifically for IMZ, may be more limited or better controlled in the latter. It should be noted that imazalil has been used since the 1970s to reduce mould growth on crops caused by species of the genus Penicillium [46]. Due to its high effectiveness, it is widely used as a fungicide, which in turn causes environmental pollution. In agricultural areas, its concentration in water can reach as high as 1 mg/L [47]. IMZ is primarily applied in agriculture to protect fruits and vegetables (including citrus fruits and potatoes) and cereals (including barley and wheat), as well as in veterinary medicine [48,49,50,51,52]. Although applied to nonedible parts, small quantities of this compound can migrate into the flesh of fruits and vegetables. Importantly, IMZ is rapidly and almost completely absorbed from the gastrointestinal tract, altering the composition of the gut microbiota [53,54]. This is particularly concerning given that gut microbiota is crucial for health, modulating digestive, nervous, and immune functions. The resulting dysbiosis in exposed individuals may create susceptibility to numerous diseases [55,56].

These findings are crucial given research on COVID-19 suggesting that alterations in the gut microbiome contribute to the pathogenesis of SARS-CoV-2. COVID-19 correlates with changes in gut microbial composition and intestinal barrier dysfunction, which may promote the translocation of bacterial products and endotoxins into the systemic circulation, thereby exacerbating systemic inflammation. Furthermore, gut dysbiosis may disrupt the gut–lung axis by impairing immune cell recruitment to the lungs, ultimately increasing susceptibility to respiratory infections [57]. In addition, Tao et al. demonstrated that IMZ induces oxidative stress, apoptosis, and cell cycle alterations in human colorectal epithelial cells (Caco-2 cells) [58]. These results suggest that IMZ exposure may not only alter gut microbiome composition but also directly damage intestinal epithelial cells, potentially compromising intestinal barrier integrity and promoting inflammatory processes.

We also observed high concentrations of four studied EDCs (MeP, PrP, 4-OP, and 4-NP) in the plasma of convalescents. Therefore, differences in the concentrations of these compounds compared with controls may be associated with prior COVID-19 status. The scale of this observation suggests significant consequences resulting from the widespread use of parabens (MeP and PrP—mainly used as antimicrobial preservatives in cosmetics, personal care products, and pharmaceuticals) [59] and phenols (4-OP and 4-NP used to manufacture surfactants, detergents, resins, adhesives, plasticizers, and antioxidants) [60,61].

The novelty of our research lies in demonstrating that a one-unit increase in the plasma concentrations of tested parabens and phenols correlates with a higher probability of prior SARS-CoV-2 infection. We observed moderate effect sizes for MeP (1.80-fold), PrP (1.70-fold), and 4-NP (1.63-fold) per unit increase, and a 300-fold odds increase per one-unit increase in 4-OP, which translates to a 1.79-fold odds increase per 0.1 unit increase. Of particular concern is 4-OP, as its strong association with immune system dysfunction underlines its potential clinical significance. In 2015, Couleau et al. demonstrated that exposure of human macrophage-like THP-1 cells to 4-OP leads to increased secretion of pro-inflammatory cytokines (interleukin (IL)-1β, IL-8, tumor necrosis factor-α) and strong inhibition of phagocytosis, which may increase the risk of COVID-19 [62]. Additionally, 4-OP was classified as a hazardous substance under Regulation (EC) No 1272/2008 and was included in the REACH Regulation toxic substances list. Subsequently, for safety reasons, the European Union restricted the use of phenol and its derivatives through European Commission directives targeting cosmetics (1223/2009) and children’s products (2009/48/EC). Despite the regulations, the persistence and bioaccumulative ability of phenolic compounds mean that trace amounts endure in the environment. Interestingly, phenol is also an antiseptic with antimicrobial properties. During the COVID-19 pandemic, this compound and its derivatives were frequently found in surface disinfectants used in hospitals and laboratories [63,64].

Recent 2024 data highlight the toxic effects of 4-OP on various human cell lines. A notable fact is that mixtures of phenols, specifically 4-OP and 4-NP, exhibit higher cytotoxicity than individual compounds. The most severe toxic effect was observed in liver cells (HepG2), where 4-OP accelerated apoptosis and disrupted cellular homeostasis [5]. These data provide a strong incentive to reconsider the use of everyday products containing these compounds.

In our exploratory ROC analysis, the model effectively discriminated prior COVID-19 status based on EDC exposure [0.7 ≤ AUC < 0.85] for 4-OP and PrP, both in the total cohort and when stratified by sex. However, those outcomes require independent external validation before they can be applied to prediction or risk classification. Additionally, a critical limitation regarding the temporal interpretation of plasma EDC concentrations must be acknowledged. The studied EDCs are unstable with relatively short biological half-lives, meaning their detection primarily reflects recent dietary, household, or occupational exposure. However, repeated daily exposure may allow these short-term EDCs to remain detectable, leading to group-level differences reflecting habitual exposure patterns.

From a broader toxicological perspective, the assessment of EDC exposure should not be limited to individual compounds. According to the European EDC-MixRisk project, humans are exposed to complex mixtures of EDCs; thus, their actual biological effects may differ from those observed for individual substances, even when individual concentrations remain within safe thresholds. This discrepancy occurs because components within EDC mixtures can exert additive or synergistic interactions, inducing biological effects that cannot be predicted by evaluating individual chemicals alone [65].

Statistical analysis indicated that approximately 25% of patients with COVID-19 had at least one comorbidity, and 8.2% had two or more. Among these comorbidities, hypertension (16.9%), diabetes (8.2%), cardiovascular disease (3.7%), and chronic kidney disease (1.3%) predominated [66,67]. Meanwhile, long-term exposure to EDCs contributes to the onset and progression of these very comorbidities [34]. Therefore, our findings in blood donors without diagnosed comorbidities shed new light on the safety of products containing these specific compounds.

**Figure 4:**
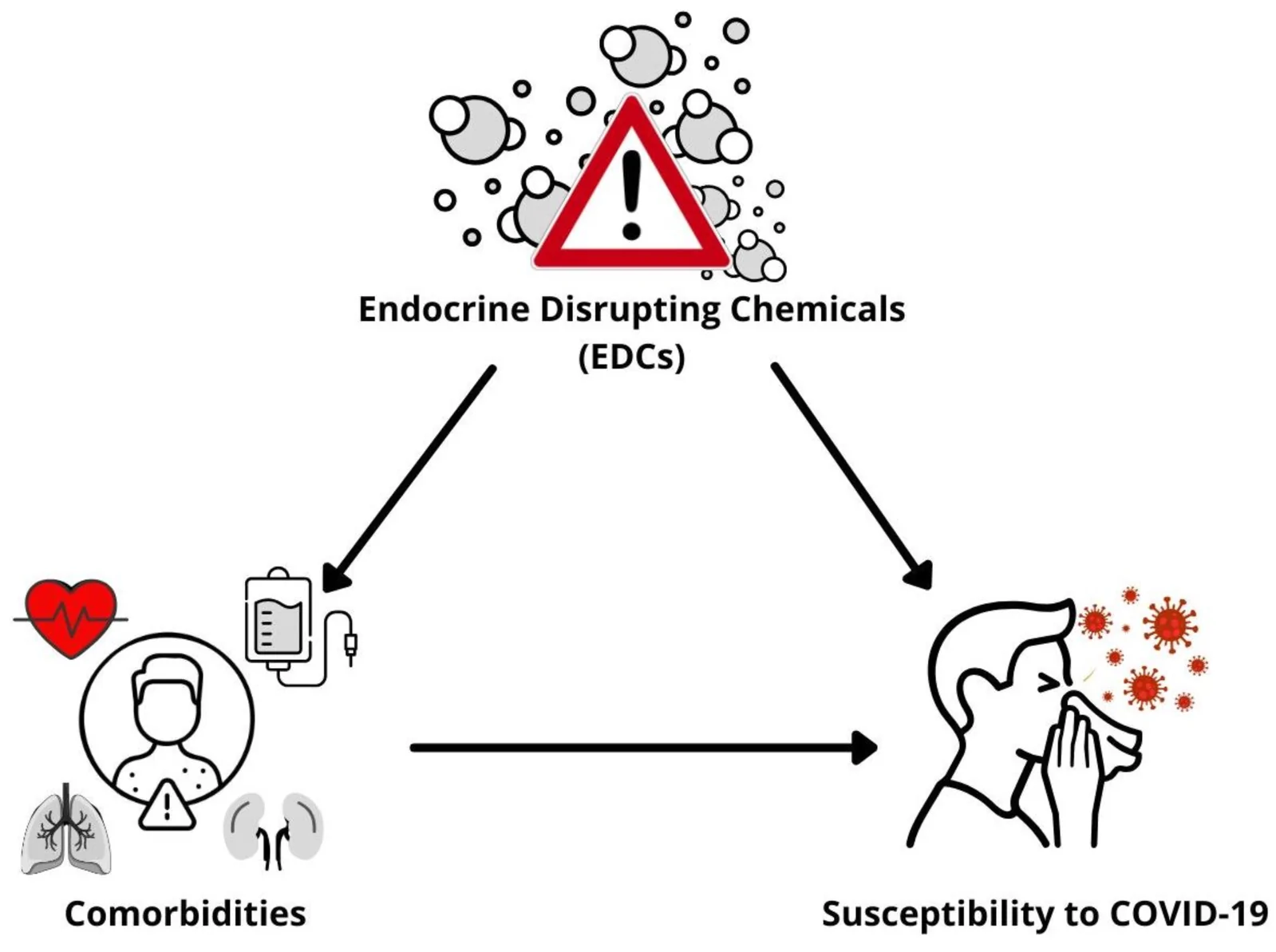
Schematic representation of the impact of EDCs on the risk of COVID-19. Source: authors’ own analysis.

Given the strong relationship between the endocrine and immune systems, EDCs may have a significant impact on immune functions [68]. When evaluating the impact of EDCs on COVID-19 risk, both their indirect effects via comorbidities and their potential direct effect must be considered (Figure 4) [69].

## 5. Conclusions

Our research focused on the relationship between the presence of EDCs in SARS-CoV-2 convalescents and healthy individuals. The results indicate that exposure to four of the seven analyzed EDCs—MeP, PrP, 4-OP, and 4-NP—may be associated with a higher likelihood of previous SARS-CoV-2 infection. Our study also identified relatively high concentrations of IMZ in the plasma of blood donors, indicating widespread and persistent exposure to this compound within the population of northeastern Poland. Given its documented ability to block the androgen receptor and interfere with testosterone, IMZ accumulation at these levels raises legitimate concerns about its potential health effects. Collectively, our results shed new light on the safety of products containing the studied compounds. Although the immediate threat of the COVID-19 pandemic has passed, the risk of future outbreaks persists; therefore, further investigation of environmental exposures as potential correlates or modifiers of SARS-CoV-2 infection history and viral infection risk.

## 6. Study Limitations

This study has several limitations. First, the sample size was relatively small, although it remained sufficient for the statistical analyses performed. Second, the study population consisted exclusively of voluntary blood donors, representing a selected and generally healthier group that may not be fully representative of the general population. Third, the dataset lacked detailed epidemiological parameters, including vaccination status and information on occupational or environmental exposure to SARS-CoV-2. In addition, data on other potentially important confounding factors, such as BMI, smoking, diet, socioeconomic status, use of personal hygiene products and disinfectants, household exposure, and time since infection, were not available. Fourth, because the eligibility of the control group relied solely on self-reported medical history and interview, undocumented inclusion of prior asymptomatic or subclinical SARS-CoV-2 infections cannot be completely ruled out. Finally, despite using rigorous statistical approaches to prevent overinterpretation, further studies involving larger and more diverse populations are warranted to validate the observed associations between selected EDC profiles and prior SARS-CoV-2 infection history.

## Figures and Tables

**Table 1:** Study group characteristics and comparison between convalescents and control group.

Variable	n	Total Group (n = 262)	Convalescents (n = 121, 46.2%)	Control Group (n = 141, 53.8%)	MD/RR (95% CI)	*p*
Gender, male, n (%)	262	214 (81.7)	110 (90.9)	104 (73.8)	1.23 (1.10; 1.38)	**0.001**
Age, years, mean ± SD	262	36.05 ± 9.67	38.38 ± 9.34	34.06 ± 9.54	4.32 (2.02; 6.63)	**<0.001**
MeP, median (IQR)	262	1.20 (0.76; 1.71)	1.39 (0.77; 1.89)	1.12 (0.72; 1.56)	0.27 (0.08; 0.45)	**0.004**
PrP, median (IQR)	246	1.38 (0.38; 2.39)	2.03 (1.06; 3.30)	0.81 (0.20; 1.89)	1.22 (0.66; 1.32)	**<0.001**
4-OP, median (IQR)	262	0.57 (0.51; 0.73)	0.69 (0.57; 1.01)	0.52 (0.47; 0.60)	0.16 (0.13; 0.23)	**<0.001**
4-NP, median (IQR)	262	1.90 (1.46; 2.42)	2.05 (1.55; 2.55)	1.78 (1.39; 2.32)	0.27 (0.07; 0.42)	**0.007**
BPA, median (IQR)	262	1.14 (0.72; 2.16)	1.34 (0.51; 3.36)	1.07 (0.77; 1.54)	0.26 (−0.12; 0.49)	0.333
DEHP, median (IQR)	262	49.68 (31.15; 89.81)	55.51 (30.98; 145.37)	47.24 (33.72; 70.47)	8.27 (−0.41; 23.13)	0.059
IMZ, median (IQR)	260	20.06 (17.90; 21.93)	20.00 (18.38; 22.71)	20.17 (17.57; 21.69)	−0.18 (−0.28; 1.49)	0.171

SD—standard deviation, IQR—interquartile range, MD—mean/median difference (convalescents vs. control group), RR—relative risk (convalescents vs. control group), CI—confidence interval, bold values indicate statistically significant differences (*p* < 0.05). Groups compared with *t*-Student test (age), Mann–Whitney U test (EDCs) or Pearson Chi-square test (gender). (Data missing for 16 PrP and 2 IMZ samples due to analytical errors).

**Table 2:** Outcome of logistic regression analysis for prior COVID-19 status.

Variable	Univariate Model			Multivariate Model		
	OR	95% CI	*p*	OR	95% CI	*p*
Gender, male (vs. female)	3.56	1.78–7.66	**0.001**	2.75	1.23–6.64	**0.018**
Age, years	1.05	1.02–1.08	**<0.001**	1.05	1.02–1.09	**0.002**
MeP	1.80	1.30–2.55	**0.001**	1.22	0.84–1.85	0.330
PrP	1.70	1.40–2.11	**<0.001**	-	-	-
4-OP	333.03	62.46–2344.47	**<0.001**	328.92	52.94–2756.22	**<0.001**
4-NP	1.63	1.19–2.28	**0.003**	-	-	-
BPA	1.07	1.01–1.16	**0.041**	-	-	-
DEHP	1.00	1.00–1.00	0.088	-	-	-
IMZ	1.06	1.00–1.14	0.055	1.08	1.01–1.17	**0.033**

OR—odds ratio, CI—confidence interval, bold values indicate statistically significant differences (*p* < 0.05).

**Table 3:** Outcomes of ROC analysis for prior COVID-19 status.

Variable	Cutoff	AUC (95% CI)	Sensitivity	Specificity	PPV	NPV	Accuracy	*p*
**Total Study Group**								
MeP	1.33	0.602 (0.535; 0.669)	0.54	0.66	0.58	0.62	0.60	**<0.001**
PrP	0.56	0.715 (0.645; 0.778)	0.87	0.45	0.58	0.80	0.65	**<0.001**
4-OP	0.55	0.809 (0.755; 0.860)	0.90	0.62	0.67	0.88	0.75	**<0.001**
4-NP	1.90	0.596 (0.528; 0.666)	0.60	0.58	0.55	0.63	0.59	**0.002**
BPA	1.74	0.535 (0.462; 0.612)	0.44	0.80	0.65	0.62	0.63	**0.009**
DEHP	143.73	0.568 (0.499; 0.640)	0.26	0.97	0.89	0.60	0.64	**0.015**
IMZ	20.08	0.451 (0.378; 0.523)	0.52	0.51	0.47	0.56	0.52	**0.0499**
**Males Only**								
MeP	1.33	0.596 (0.522; 0.673)	0.53	0.67	0.63	0.57	0.60	**0.003**
PrP	2.39	0.701 (0.624; 0.770)	0.42	0.91	0.83	0.60	0.66	**<0.001**
4-OP	0.55	0.803 (0.743; 0.859)	0.91	0.60	0.70	0.86	0.76	**<0.001**
4-NP	1.90	0.591 (0.515; 0.664)	0.59	0.59	0.60	0.58	0.59	**0.011**
BPA	1.74	0.555 (0.476; 0.634)	0.44	0.84	0.74	0.58	0.63	**0.006**
DEHP	71.56	0.568 (0.488; 0.645)	0.45	0.79	0.69	0.57	0.61	**0.045**
IMZ	22.64	0.583 (0.507; 0.657)	0.27	0.91	0.76	0.55	0.58	**0.010**
**Females Only**								
MeP	2.84	0.617 (0.405; 0.808)	0.27	1.00	1.00	0.82	0.83	**0.023**
PrP	0.84	0.718 (0.556; 0.866)	0.82	0.62	0.41	0.91	0.67	**0.042**
4-OP	0.54	0.824 (0.667; 0.953)	0.91	0.65	0.43	0.96	0.71	**<0.001**
4-NP	2.35	0.639 (0.437; 0.830)	0.55	0.78	0.43	0.85	0.73	**0.048**
BPA	1.57	0.474 (0.246; 0.720)	0.55	0.68	0.33	0.83	0.65	0.243
DEHP	49.63	0.496 (0.256; 0.717)	0.55	0.65	0.32	0.83	0.62	0.150
IMZ	20.35	0.631 (0.403; 0.843)	0.73	0.62	0.36	0.88	0.65	0.165

AUC—area under curve, CI—confidence interval, PPV—positive predictive value, NPV—negative predictive value, bold values indicate statistically significant differences (*p* < 0.05).

## Data Availability

The datasets used and/or analyzed during the current study are available from the corresponding author on reasonable request. Data will be made available on request.

## Supplementary Materials

The following supporting information can be downloaded at: https://www.mdpi.com/article/10.3390/life16081379/s1. Supplementary Table S1. Outcome of multiple-testing correction for group comparisons and univariable logistic regression analyses. Supplementary Table S2. Outcome of univariate logistic regression models performed as sensitivity analyses for association between 4-OP and prior COVID-19 status. Supplementary Table S3. Outcome of multivariate logistic regression for prior COVID-19 infection performed as sensitivity analyses (no pre-selection of covariates).
