# Supplementary material for: Environmental Exposure to Endocrine-Disrupting Chemicals and SARS-CoV-2 Infection History Among Blood Donors
Source: Life (Basel). 2026 Aug 21;16(8):1379. doi: 10.3390/life16081379 (PMC13514347; doi:10.3390/life16081379)
Supplement: Supplementary file 1 [file life-16-01379-s001.zip › life-4321342-supplementary.pdf]

Supplementary Table S1. Outcome of multiple-testing correction for group comparisons and univariable logistic regression analyses

**Variable**	**Comparison between convalescents and control group**		**Univariate logistic regression for prior COVID-19 status**	
	**p (uncorrected)**	**p (FDR)**	**P (uncorrected)**	**p (FDR)**
Gender, male (vs female)	**0.001**	**0.001**	**0.001**	**0.001**
Age, years	**<0.001**	**0.001**	**<0.001**	**0.001**
MeP	**0.004**	**0.006**	**0.001**	**0.001**
PrP	**<0.001**	**<0.001**	**<0.001**	**<0.001**
4-OP	**<0.001**	**<0.001**	**<0.001**	**<0.001**
4-NP	**0.007**	**0.010**	**0.003**	**0.005**
BPA	0.333	0.333	**0.041**	0.053
DEHP	0.059	0.071	0.088	0.088
IMZ	0.171	0.187	0.055	0.062

FDR – Benjamini-Hochberg false discovery rate correction. FDR-corrected p-values were calculated separately for the group comparison analyses and the univariable logistic regression analyses.

Supplementary Table S2. Outcome of univariate logistic regression models performed as sensitivity analyses for association between 4-OP and prior COVID-19 status

**Variable**	**n**	**OR**	**95% CI**	**p**
4-OP: per 1-unit increase (original)	262	333.03	62.46-2344.47	**<0.001**
4-OP: per 0.1 unit increase	262	1.79	1.51-2.17	**<0.001**
4-OP: per 1-unit increase excl. outliers *	232	12393.73	807.52-290882.75	**<0.001**
4-OP: per 0.1 unit increase excl. outliers *	232	2.57	1.95-3.52	**<0.001**

OR – odds ratio, CI – confidence interval.

* Outliers defined as observations observations exceeding the upper Tukey fence (Q3 + 1.5 × IQR).

Supplementary Table S3. Outcome of multivariate logistic regression for prior COVID-19 infection performed as sensitivity analyses (no pre-selection of covariates)

**Variable**	**OR**	**95% CI**	**p**
Gender, male (vs female)	2.29	0.99-5.69	0.061
Age, years	1.06	1.02-1.10	**0.001**
MeP	1.51	0.96-2.54	0.101
PrP	1.19	0.90-1.61	0.227
4-OP (per 0.1 unit increase)	1.68	1.33-2.18	**<0.001**
4-NP	0.74	0.45-1.20	0.234
BPA	1.03	0.98-1.12	0.387
DEHP	1.00	1.00-1.00	0.371
IMZ	1.06	0.99-1.15	0.112

OR – odds ratio, CI – confidence interval.
